# Recent Advances in Ruddlesden–Popper Phase-Layered Perovskite Sr_2_TiO_4_ Photocatalysts

**DOI:** 10.3390/nano15010020

**Published:** 2024-12-27

**Authors:** Pei Wang, Lijun Liao, Hongqi Chu, Ying Xie, Zhenzi Li, Wei Zhou

**Affiliations:** 1 Shandong Provincial Key Laboratory of Molecular Engineering, School of Chemistry and Chemical Engineering, Qilu University of Technology (Shandong Academy of Sciences), Jinan 250353, China; pei_wang00@163.com (P.W.); lijunliao@qlu.edu.cn (L.L.); hqchu@qlu.edu.cn (H.C.); zzli@qlu.edu.cn (Z.L.); 2 Key Laboratory of Functional Inorganic Material Chemistry, Ministry of Education, School of Chemistry and Materials Science, Heilongjiang University, Harbin 150080, China; xieying@hlju.edu.cn

**Keywords:** photocatalysis, Sr_2_TiO_4_, Ruddlesden-Popper perovskite, layered structure, solar energy conversion

## Abstract

Sr_2_TiO_4_, a prominent member of the Ruddlesden–Popper (RP) perovskite family, has garnered significant interest in photocatalysis, primarily owing to its distinctive two-dimensional (2D) layered structure. In this review, we provide an insightful and concise summary of the intrinsic properties of Sr_2_TiO_4_, focusing on the electronic, optical, and structural characteristics that render it a promising candidate for photocatalytic applications. Moreover, we delve into the innovative strategies that have been developed to optimize the structural attributes of Sr_2_TiO_4_. These strategies aim to maximize light absorption, improve charge separation, and accelerate the photocatalytic reaction rates. By highlighting these unique approaches, we strive to contribute to a more profound understanding of the material’s potential and stimulate further advancements in developing Sr_2_TiO_4_-based photocatalytic systems. The review not only synthesizes the existing knowledge but also offers a perspective in future directions for research and application. As the field of photocatalysis continues to evolve, Sr_2_TiO_4_ stands poised to play a pivotal role in the quest for more efficient and sustainable solar energy conversion technology.

## 1. Introduction

With the increasingly severe problems of energy shortage and environmental pollution, the development of green renewable energy is highly urgent [1,2,3,4,5]. Photocatalytic technology can convert low-density solar energy into high-density chemical energy, and it has been widely applied in the degradation of pollutants and the production of hydrogen energy [6,7,8,9]. In the photocatalytic process, the absorption of visible light and the efficiency of photogenerated electron–hole pair separation emerge as critical factors. An appropriate photocatalyst can enhance visible light absorption and photogenerated carrier separation, thereby improving photocatalytic performance [10,11,12,13]. Amongst other factors, due to the rich elemental composition, high chemical stability, and flexible band structures and optical properties, perovskite oxides and their derivatives are considered to be promising photocatalysts [14,15]. For example, Takata et al. studied the Al-doped SrTiO_3_ photocatalyst that exhibited outstanding performance and introduced an ideal cocatalyst structure for effective water splitting without charge recombination loss [16].

**Figure 1:**
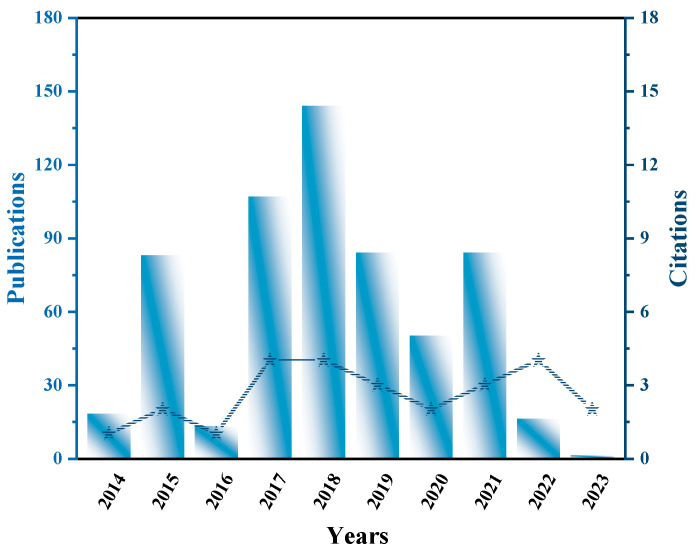
Publication and citation data on Sr_2_TiO_4_ photocatalysis in recent years.

The chemical formula of perovskite is ABX_3_, where A and B are cations, and X is an anion. Taking perovskite oxides as an example, under ideal conditions, a single perovskite oxide (ABO_3_) possesses a cubic structure. It consists of a flexible framework formed by chains of corner-sharing [BO_6_] octahedra, in which the A-site cations occupy the cavities in a cubic octahedral symmetry [17]. It has been reported that the performance of original 3D perovskites can be tuned by introducing structural distortions to adjust their electronic, physical, electrocatalytic, and photocatalytic properties [18,19]. In other words, three-dimensional (3D) perovskite materials can be transformed into one-dimensional (1D) and two-dimensional (2D) perovskite oxides, which are considered perovskite oxides with lower symmetry [20]. Compared to the traditional ABO_3_ perovskites, the unique layered structure of 2D Ruddlesden–Popper (RP) perovskites is particularly noted for its ability to inhibit carrier recombination and promote electron transfer, thus receiving special attention [21,22,23]. As a representative of RP phase perovskite, Sr_2_TiO_4_ is a promising photocatalyst for HER, OER, and pollutant degradation, which has attracted the attention of researchers due to its unique optical, structural, and electronic properties (Figure 1) [24,25,26]. Compared with other photocatalysts such as TiO_2_ and BiVO_4_, Sr_2_TiO_4_ has a unique layered crystal structure, and each two layers of SrO and TiO_2_ are connected by oxide chains. The layered structure allows carriers to migrate along the crystal plane, which improves the migration efficiency of photogenerated carriers. In addition, the layered structure provides a larger specific surface area and provides more active sites for the catalytic reaction. The charge transfer is confined to a single octahedral layer, and the interlayer transfer across adjacent layers is not allowed. This charge transfer characteristic of two-dimensional plays an important role in the separation of photocatalytic carriers [26]. For example, Zadeh et al. prepared nanostructured Sr_2_TiO_4_ by a simple chemical synthesis method and studied its photocatalytic performance for the degradation of toxic dyes in aqueous solution. Studies have shown that nano-Sr_2_TiO_4_ has high photocatalytic efficiency, good catalytic stability and visible light response ability, showing its potential in environmental governance [25]. However, the wide band gap (~3.5 eV) and poor photocatalytic activity under visible light of the original Sr_2_TiO_4_ limit its application [21]. In order to further optimize the structural characteristics and photocatalytic performance of Sr_2_TiO_4_, numerous modification strategies have been studied [17]. Sun et al. optimized the photocatalytic performance of Sr_2_TiO_4_ by co-doping La and N, and significantly improved its hydrogen production efficiency under visible light [26]. Hu et al. constructed a Ag@Sr_2_TiO_4_/Bi_5_O_7_I heterojunction, which significantly facilitated the separation efficiency of electrons and holes as well as improved the photocatalytic hydrogen generation rate [27].

**Figure 2:**
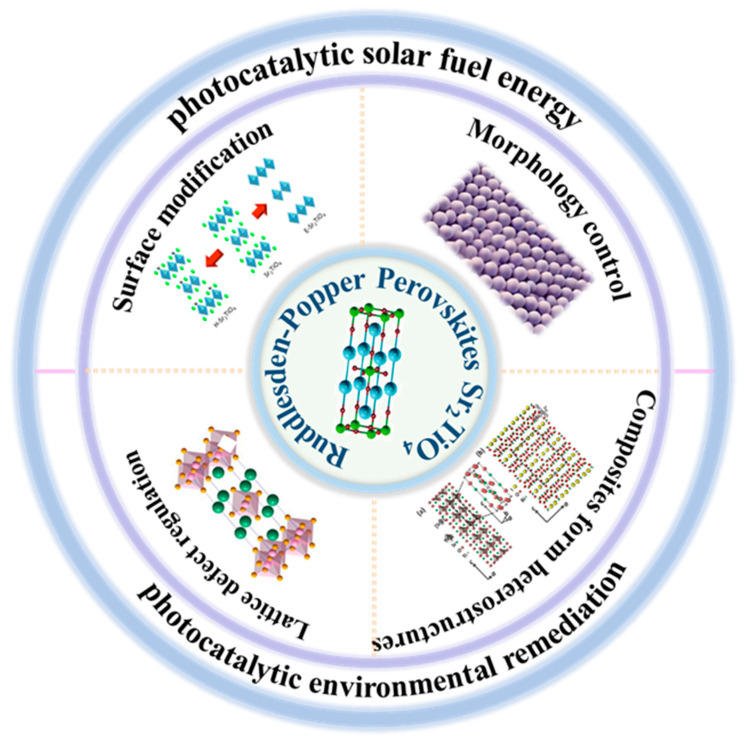
Schematic diagram of the main content of this review.

Although an excellent review on RP-structured perovskite photocatalysts has been published [17], to our knowledge, no review on Sr_2_TiO_4_ has been reported. Even though Sr_2_TiO_4_ shows great application potential, a fundamental understanding of the influence of its structure and electronic properties on photocatalytic activity is still lacking. This review aims at summarizing the recent advances in the design and development of RP perovskites Sr_2_TiO_4_ as photocatalysts for pollutant degradation, hydrogen production and CO_2_ reduction and presents a perspective on the future investigation of Sr_2_TiO_4_ layered perovskites. First, we introduce the structure of Sr_2_TiO_4_ layered perovskites and their unique characteristics for photocatalysis-based applications. Then, the recent advances in the design and development of RP perovskites Sr_2_TiO_4_ for photocatalysis are summarized and discussed by providing design strategies (Figure 2). We emphasize the targeted introduction of dopants at single or double lattice sites, which have been shown to significantly alter the electronic properties of the material. Additionally, the formation of composites with other materials, surface modification and morphology control have been discussed for its potential to expand the light absorption capabilities and augment the catalytic activity of Sr_2_TiO_4_. Finally, current challenges and future directions in the development of Sr_2_TiO_4_ layered photocatalysts are discussed.

It is gratifying to observe that the findings presented in this review offer valuable insights and inspiration for the future development of Sr_2_TiO_4_. The innovative strategies discussed herein hold the potential to significantly contribute to the advancement of layered Sr_2_TiO_4_ photocatalysts, pushing the boundaries of their applications in environmental remediation and renewable solar energy production. The review encapsulates the current state of knowledge, emphasizing the importance of continued exploration into the material’s properties and the development of novel modification methods. As the field of photocatalysis progresses, Sr_2_TiO_4_ is poised to play a pivotal role in the quest for more efficient and sustainable solar energy conversion technology.

## 2. The Properties and Modification of Sr_2_TiO_4_

### 2.1. Sr_2_TiO_4_

**Figure 3:**
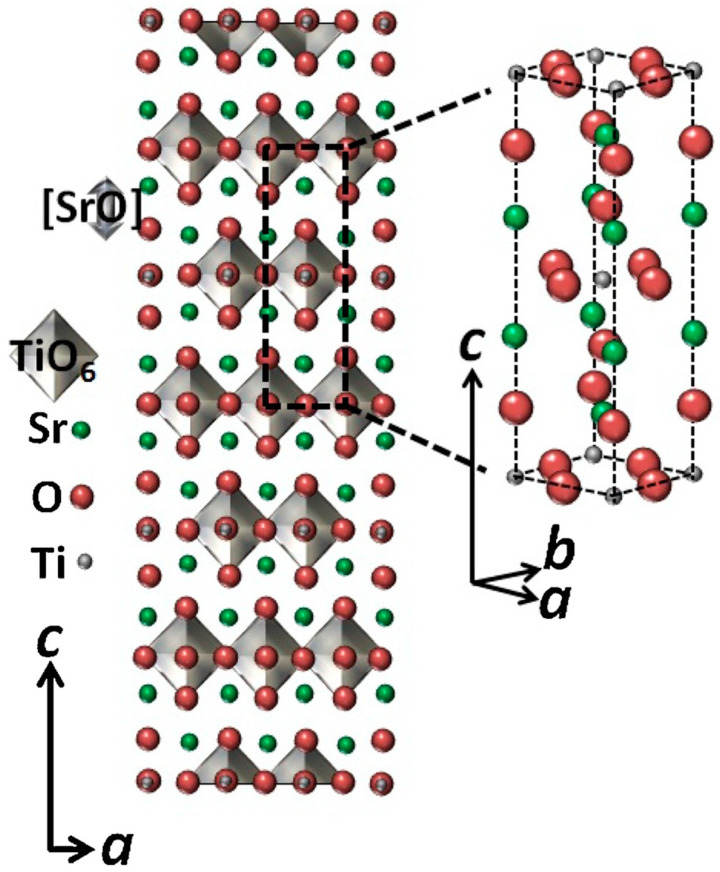
Refined crystal structures and unit-cells of Sr_2_TiO_4_. The dashed rectangles in the crystal structure represent the a × c facet of the corresponding unit cells [27].

Sr_2_TiO_4_ is a wide band gap semiconductor, which is similar to TiO_2_ [28]. The space group is I4/mm, and the cell parameters are a = 3.884 Å and c = 12.6 Å [29]. In the 2D layered perovskite structure of Sr_2_TiO_4_, Ti atoms are located in a six-coordinate octahedral environment, the TiO_6_ octahedra layer and the SrO rock salt layer are alternately stacked along the c-axis, and its crystalline structure is shown in Figure 3 [27,30]. Owing to the special layered crystal structure and typical 2D charge transport characteristics, which are conducive to the more effective separation of photogenerated electrons and holes, Sr_2_TiO_4_ exhibits more excellent photocatalytic performance than perovskite SrTiO_3_ [26,31].

However, the wide band gap of Sr_2_TiO_4_ shows photocatalytic activity only under light with a wavelength of λmax = 253 nm, seriously affecting the quantum yield and photocatalytic response in a narrow spectral range [32,33]. Therefore, in developing effective photocatalysts with extended optical range, controlling the band gap of semiconductors is still a priority [34]. To this end, researchers have also developed many modification strategies to expand the light absorption range in such materials [35].

### 2.2. Modification Strategies

#### 2.2.1. Surface Modification

It is well known that the catalytic reaction is a chemical reaction on the surface, so the catalytic activity can be significantly improved by changing the surface properties of the catalyst [36,37]. In photocatalysis, the surface of the catalyst can easily provide a site for the compositing of photogenerated electrons (e^−^) and holes (h^+^) that undergo redox reactions [38,39]. Therefore, the surface physical and chemical properties of the catalyst play a decisive role in its photocatalytic performance [40,41].

The surface modification of Sr_2_TiO_4_-based photocatalytsts can be achieved via the treatment of the surface of the material by various physical, chemical or mechanical means to make it have specific functions or properties, such as defect engineering, cocatalysts deposition, etc. [42]. Gorkusha et al. introduced oxygen vacancies into Sr_2_TiO_4_ by using water-cooled iron barrel low-temperature reduction technology, and they established the relationship between the planar defect concentration and the actual chemical composition (non-stoichiometric) of the Sr_2_TiO_4_ phase. Studies have shown that the presence of defects leads to the enrichment of Ti in the particle volume, thereby enriching the surface Sr and optimizing the catalytic activity of Sr_2_TiO_4_ in the oxidative coupling of methane [43]. In the study of Shao et al., a facile method to reduce Sr_2_TiO_4_ in hydrogen and argon atmosphere can encourage some oxygen atoms to separate from the lattice and form oxygen vacancies [44]. Oxygen vacancies can be used as electron donors to increase the carrier concentration of semiconductors, thereby improving charge transport capacity. Moreover, the increased oxygen vacancies provide more active sites, which is conducive to the photocatalytic reaction. In order to improve the photocatalytic activity of the photocatalysts, noble metal such as silver (Ag) is widely used as a cocatalyst because of its unique localized surface plasmon resonance (LSPR) and high conductivity [27]. Hu et al. prepared Ag-loaded Bi_5_O_7_I/Sr_2_TiO_4_ heterostructure composites by photodeposition on the surface of Sr_2_TiO_4_ and Bi_5_O_7_I. The analysis shows that the loading of Ag on the surface of Bi_5_O_7_I/Sr_2_TiO_4_ heterostructure induces localized surface plasmon resonance and increases the visible light absorption, thus promoting the photocatalytic degradation of MO solutions [27].

**Figure 4:**
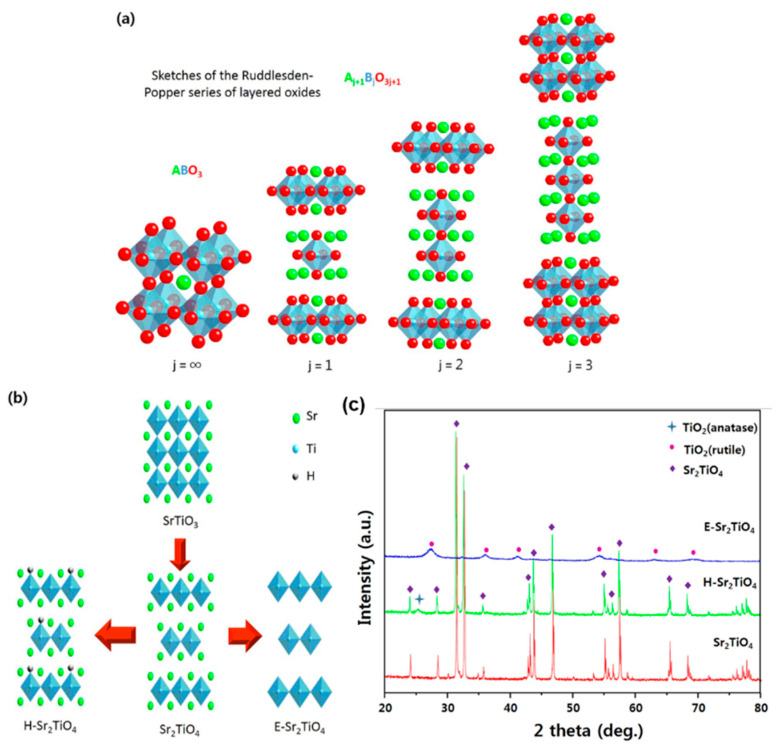
(**a**) Ruddlesden–Popper phase of layered oxide, (**b**) the overview of catalyst surface modification and (**c**) XRD patterns of Sr_2_TiO_4_, H-Sr_2_TiO_4_ and exfoliation of Sr_2_TiO_4_ catalysts [45].

**Figure 5:**
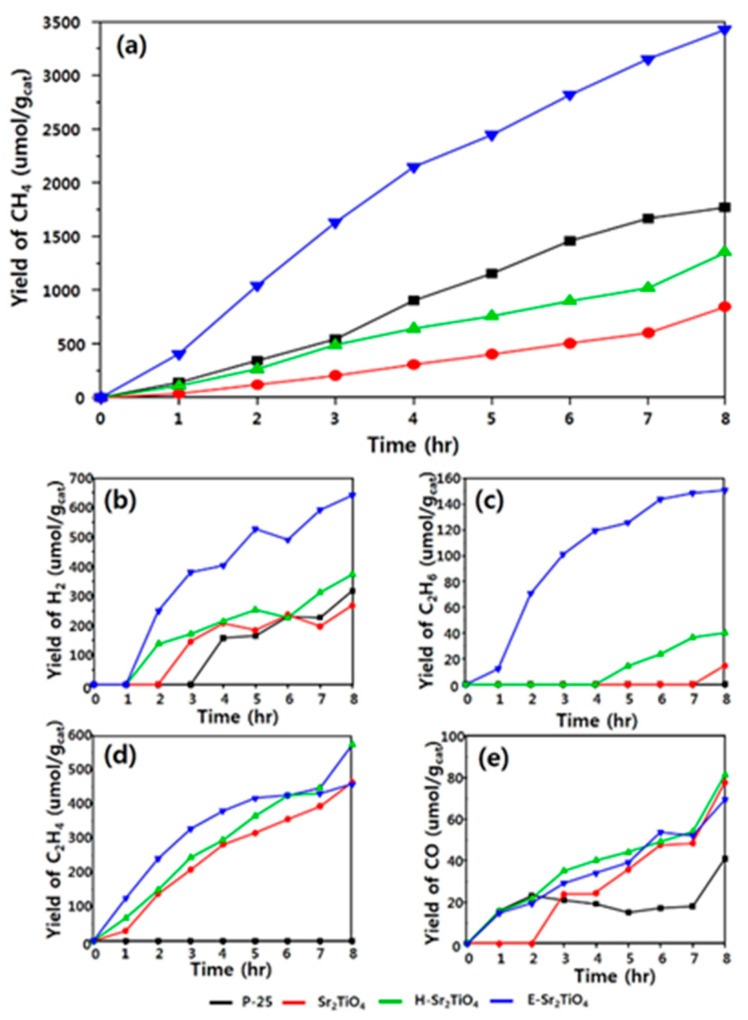
Photocatalytic CO_2_ reduction with H_2_O on catalysts. (**a**) yield of CH_4_, (**b**) yield of H_2_ (**c**) yield of C_2_H_6_ (**d**) yield of C_2_H_4_, and (**e**) yield of CO [45].

In addition, the surface modification can be carried out to increase the surface area of layered perovskite Sr_2_TiO_4_ and thus improve its photocatalytic activity for CO_2_ reduction. Kwak et al. synthesized the layered perovskite Sr_2_TiO_4_ photocatalyst by using the sol–gel method with citric acid and modified it via a unique exfoliation technique. In the exfoliation process, Sr and H ions were first ultrasonically ion-exchanged in 1 M HNO_3_ for 5 days and then ultrasonically treated in tetrapropylammonium hydroxide (TPAOH) for 3 weeks. This process destroyed part of the Sr_2_TiO_4_ structure, resulting in the separation and exposure of the internal TiO_2_ layer (Figure 4) [45]. The exfoliated Sr_2_TiO_4_ catalyst (E-Sr_2_TiO_4_) possesses a larger surface area (358.54 m^2^/g), which is about 300 times that of the original Sr_2_TiO_4_. Furthermore, E-Sr_2_TiO_4_ exhibits a narrow band gap and high dispersion, and the exposed TiO_2_ not only serves as the main contributor to light absorption and charge generation but also enhances the overall performance of photocatalytic CO_2_ reduction by synergistic effect with Sr_2_TiO_4_ (Figure 5).

#### 2.2.2. Morphology Control

In addition to the surface properties, the morphology of the catalysts also affects their photocatalytic activity [46]. Accurately controlling the morphology to optimize its structural, optical, and electrical properties is critical for evaluating the shape-dependent photoreactivity and developing high-performance photocatalysts, such as increasing surface area and pore volume, enhancing light absorption, and improving photogenerated charge separation and transfer efficiency [47,48]. There are many factors affecting the morphology and size of the catalyst, including the synthesis route, synthesis conditions, calcination temperature and so on (Table 1).

For example, Sun et al. prepared Sr_2_TiO_4_ nanoparticles (200 nm) by the polymerization complexation method at a calcination temperature as low as 900 °C [21]. Such a small grain size is highly beneficial for photocatalytic reactions not only because of a large surface area and hence more reaction sites but also due to a short charge migration pathway from bulk to the surface. In the research of Hu et al., a sol–gel method was used to synthesize a Sr_2_TiO_4_ sample that consisted of particles with cubic and rod-like morphologies and sizes of 3–6 μm [27]. After coupling the low content of Bi_5_O_7_I, the bulk structure of Sr_2_TiO_4_ was not changed. However, the Bi_5_O_7_I particles with the size of several hundred nanometers grow on the surface of Sr_2_TiO_4_, which leads to an increase in the surface roughness. At the interface, a space charge region is formed owing to the p–n junction in the Bi_5_O_7_I/Sr_2_TiO_4_ heterostructure, promoting effective charge separation and outstanding photocatalytic performance. Vijay et al. combined the polymer method and the hydrothermal method to synthesize Sr_2_TiO_4_ by calcining at 650 °C and then calcining at 1000 °C for 12 h, which showed a nanosheet morphology with a size of 250 nm [49]. In the absence of any cocatalyst, the Sr_2_TiO_4_ nanosheets exhibit a hydrogen production performance of 22.6 μmol.

Isupvoa and his colleagues used four different preparation routes to synthesize Sr_2_TiO_4_ [50]. Among them, the samples prepared by the co-precipitation (CO) method formed circular particles of about 4.7 microns, showing excellent sintering characteristics. Moreover, the sintered particles with better crystal structure were formed by the citrate precursor (CT) method, indicating that the crystallinity was improved. Additionally, the particle size of the samples prepared by mechanochemical (MA) methods is 2–10 microns with a rough surface, which may be heterogeneous. In the co-precipitation method, the different types of precipitants also affect the morphology of the catalyst. In the process of sol precipitation (SP), the different types of precipitants also affect the morphology of the catalyst. The samples prepared with (NH_4_)_2_CO_3_ as the precipitant were composed of 30–90 nm spherical nanoparticles and aggregated into large aggregates with a diameter of 5 microns. The prepared sample comprises irregularly shaped flat particles of 1.9–5.7 microns, which used K_2_CO_3_ as a precipitant.

Compared with the traditional high-temperature calcination method, the sono-chemical method can be carried out under mild conditions, and ultrasonic wave energy is used to promote the chemical reaction to achieve efficient energy transfer. In addition, ultrasound can effectively disperse the reactants and prevent particle agglomeration, thereby obtaining uniform nanostructures. In the sono-chemical method, the choice of alkaline agent is the key factor affecting the morphology of the catalyst. Different alkaline agents (such as ammonia, ethylenediamine, propylenediamine, etc.) will significantly affect the morphology and particle size of the product.

**Figure 6:**
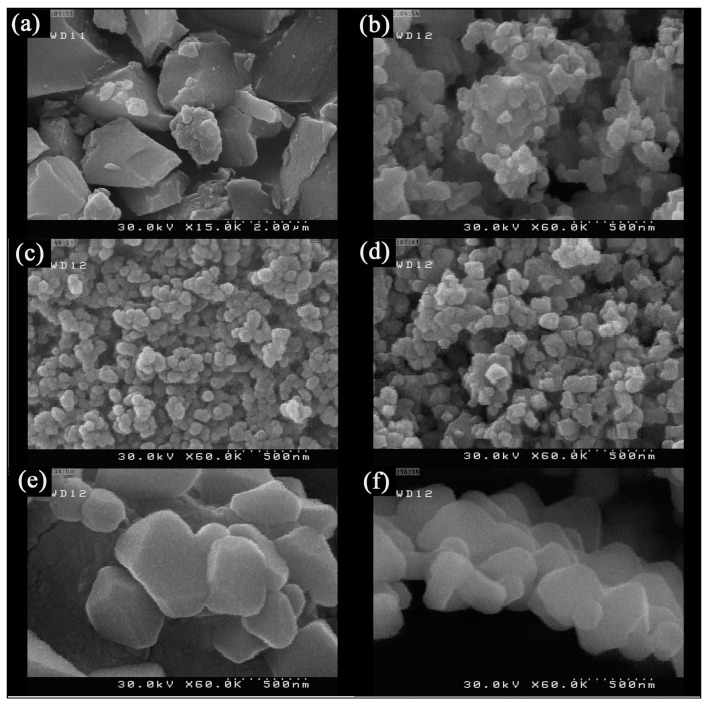
SEM images of sample No. (**a**) 1 (**b**) 2 (**c**) 3 (**d**) 4 (**e**) 5 (**f**) 6 [25].

In the research of Zadeh and his coworkers, Sr_2_TiO_4_ with various morphology structures were synthesized via a sono-chemical method by adjusting the number of amine groups of alkaline agents (Figure 6) [25]. The as-prepared Sr_2_TiO_4_ has macrostructures with the size of 2 μm in the absence of any alkaline agent. Nanostructures are obtained in the presence of NH_3_ as an alkaline agent, while the size and morphology of the products are not uniform. The morphology and size distribution were uniformly improved using ethylene diamine as an alkaline agent. Moreover, nanoparticles were prepared by changing the alkaline reagent from ethylenediamine to propylene diamine, but the size of these nanoparticles appeared to increase. By increasing the number of amino groups and using triethylenetetramine as an alkalizer, the size and morphology of the product were changed to form a quasi-geometric structure. However, the size of the product has changed, and the quasi-geometric structure tends to be interlocked by using tetra ethylene pentamine.

**Figure 7:**
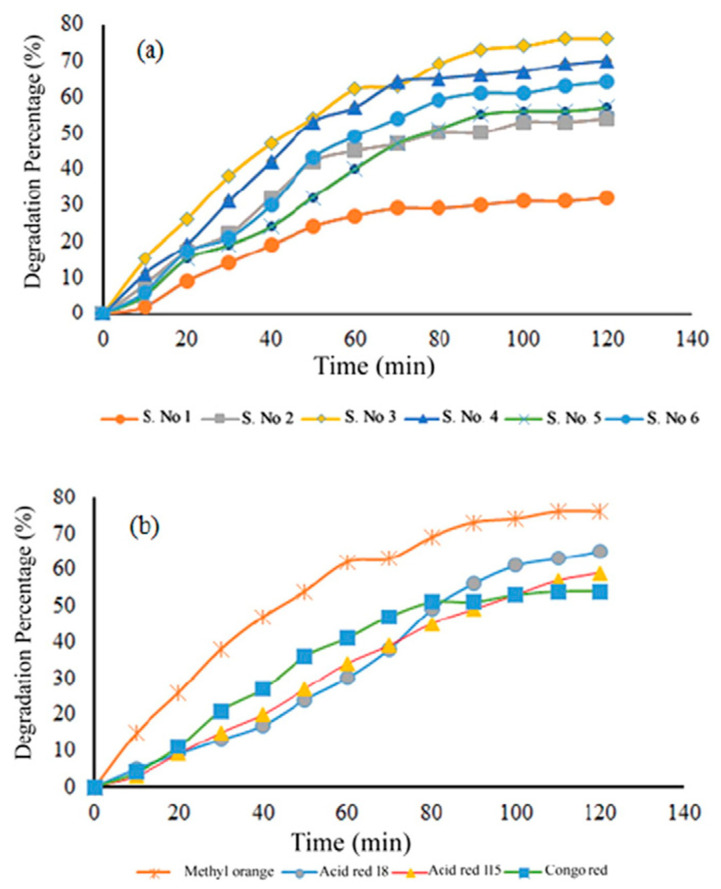
Degradation diagrams of (**a**) methyl orange on presence of different Sr_2_TiO_4_ (**b**) different dyes with sample No. 3 [25].

Furthermore, the photocatalytic activity of photocatalysts with different morphologies was studied for the degradation of azo dyes as models of chemical contaminants. It is reported that compared with the macrostructure and quasi-geometrical structure, the nanostructure exhibits more outstanding photocatalytic performance (Figure 7). This is due to the smaller and more uniform nanoparticles having a higher surface area, which is conducive to the photocatalytic reaction, thereby improving the photocatalytic efficiency.

#### 2.2.3. Lattice Defect Regulation

The regulation of lattice defects is one of the critical methods to improve photocatalytic performance by changing the crystal structure of photocatalytic materials and optimizing their electronic and surface properties [52]. Adjusting the lattice defects can significantly optimize the band gap, the separation efficiency of electron–hole pairs, and the number of surface-active sites [53].

Currently, functional doping is the most widely used lattice defect regulation strategy of Sr_2_TiO_4_ [54,55,56,57]. The doping at different sites will have different effects on the structure of Sr_2_TiO_4_. Specifically, doping at A (Sr) sites usually changes the carrier concentration by introducing additional electrons or holes and may also cause lattice distortion, thus affecting electron mobility. For instance, Xiao et al. developed a simple approach of Ag doping and reducing pretreatment to tune the properties of Sr_2_TiO_4_ photocatalysts [44]. The synergistic effect of Ag doping and moderate reduction pretreatment endows the Sr_2_TiO_4_ photocatalyst with improved morphology, increased specific surface area and oxygen vacancies, optimized light absorption capacity and band gap, and suppressed charge carrier recombination. Benefiting from these advantages, r-Ag_0.05_Sr_1.95_TiO_4_ with appropriate Ag doping and moderate reduction pretreatment exhibits excellent photocatalytic hydrogen production performance. The average hydrogen production rates under full light and visible light irradiation are 1695 mmol h^−1^ g^−1^ and 541 mmol h^−1^ g^−1^, respectively.

B (Ti) site doping is the most direct position to adjust the electronic structure and band gap. Doping elements can directly interact with the conduction band and valence band of TiO_2_, introduce new energy levels, reduce the effective band gap, and enhance the absorption of visible light. In the study of Yun and his coworkers, the band gap of Sr_2_Sc_0.125_Ti_0.875_O_4_ is narrower than that of Sr_2_TiO_4_, and the dispersion of the conduction band and valence band of Sr_2_Sc_0.125_Ti_0.875_O_4_ is enhanced, which is beneficial to improving the photocatalytic performance [58]. Similarly, another research study of this group demonstrated that the valence bands (VBs) of the Sr_2_In_0.125_Ti_0.875_O_4_ are composed of O 2*p* and In 4*d* states, which makes the top VBs shift significantly to high energies, expanding the absorption of visible light, which has practical significance for the application of Sr_2_TiO_4_ as photocatalyst [59].

**Figure 8:**
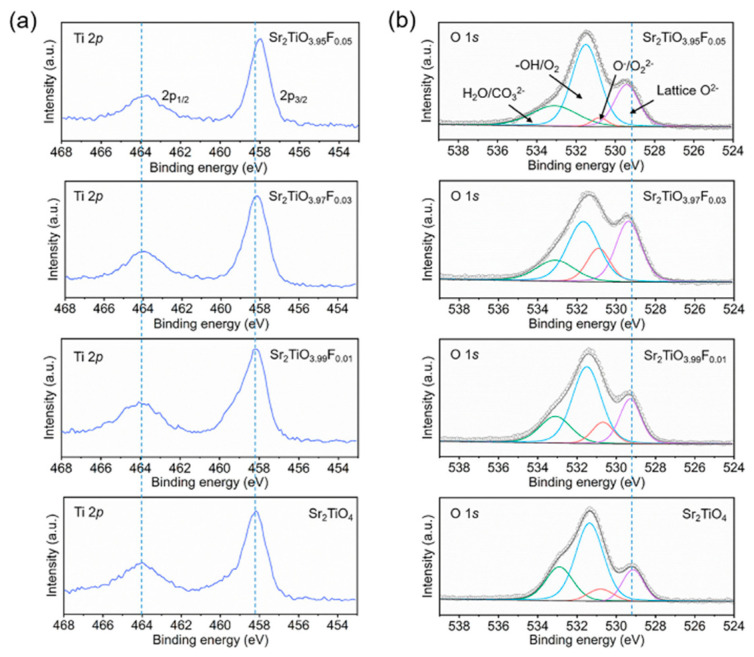
XPS spectra of Sr_2_TiO_4−x_F_x_ (x = 0, 0.01, 0.03 and 0.05): (**a**) Ti 2p, (**b**) O 1s [51].

**Figure 9:**
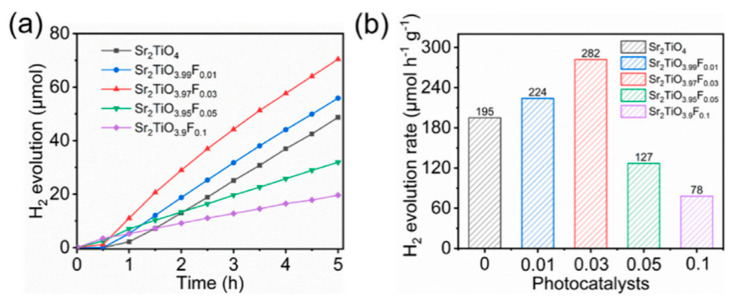
The H_2_ production (**a**) amounts and (**b**) rates of solar water splitting on Sr_2_TiO_4-x_F_x_ (x = 0, 0.01, 0.03, 0.05, and 0.1) under full-range sunlight illumination (λ ≥ 250 nm) [51].

Generally, the doping at oxygen sites will change the electron affinity and ionization energy of Sr_2_TiO_4_, thus affecting the band gap. Ziati et al. studied the effect of different concentrations of S, Se, and Te elements on the structural and optical properties of Sr_2_TiO_4_. They calculated the formation energies of each doped structure to examine the feasibility of the synthesis [53]. The results show that an increased concentration of chalcogenide elements decreases the band gap significantly, and the absorption capacity is enhanced under visible light. Furthermore, an appropriate amount of oxygen vacancies can be introduced into the material as an electron capture center to improve the separation efficiency of electron–hole pairs. Han et al. introduced appropriate surface oxygen vacancies by optimizing the F doping amount to inhibit the recombination of charge carriers (Figure 8), thus significantly improving the photocatalytic hydrogen production efficiency [51]. According to deconvolution results of O 1s, the peaks at 530.8 eV represent the O_2_^2−^/O^−^ species, which were closely related with the surface oxygen vacancies. The suitable increase in the amount of surface oxygen vacancies as an electron donor can improve the charge carrier transport capability, benefiting for the photocatalytic performance [51]. The photocatalyst of Sr_2_TiO_3.97_F_0.03_ achieved a 44% larger rate of H_2_ generation (282 mmol/h/g) than that of Sr_2_TiO_4_ (195 mmol/h/g) (Figure 9).

In addition to the above single-site doping, there are also double-sites doped, and the properties of Sr_2_TiO_4_ doped with different ions are shown in Table 2 Significantly, co-doping has attracted more interest because it can maintain charge balance without forming oxygen vacancies [32]. Sun et al. reported that introducing Cr can effectively improve its absorption of visible light by narrowing the band gap of Sr_2_TiO_4_. Nevertheless, the single doping of Cr will result in the formation of harmful species Cr^6+^, and co-doping with La can inhibit the formation of this species due to the charge balance of La [24]. The ion sizes of Rh^3+^ and Cr^3+^ in octahedral coordination are similar, and the dispersion of the Rh 4d orbital is much larger than that of the Cr 3d orbital, which means that Rh may be a good dopant for Sr_2_TiO_4_ in prolonging visible light absorption and optimizing photocatalytic activity. Based on the previous research of La/Cr co-doping, the research group introduced La/Rh into Sr_2_TiO_4_, and the band gap of Sr_2_TiO_4_ was significantly reduced due to the formation of a new valence band of Rh 4d, improving the photocatalytic hydrogen production significantly [21]. Obviously, Cr and Rh are usually the most effective dopants to improve the photocatalytic performance [60,61]. However, the harmfulness of Cr and the scarcity of Rh fundamentally hinder their wide application. Therefore, environmentally friendly and abundant dopant Fe species have attracted great interest in achieving the target of doping strategy. Zhang and his colleagues reduced the band gap of Sr_2_TiO_4_ by doping an appropriate amount of La/Fe, greatly improved the optical absorption in the visible light region, and significantly enhanced photocatalytic hydrogen evolution [31].

As is well known, O and N have similar structural, electronic, and chemical properties. The band structure of Sr_2_TiO_4_ can be effectively adjusted by N^3−^ substituting O^2−^. Wang et al. selected N to dope Sr_2_TiO_4_ and adjusted the band structure by replacing O^2−^ with N^3−^. However, the oxygen atoms in Sr_2_TiO_4_ were negatively charged, and nitrogen doping introduced additional electrons in the crystal structure. In order to maintain the overall charge neutrality, some oxygen atoms may be removed to form oxygen vacancies, and too high oxygen vacancy concentration will become a carrier recombination center, which is not conducive to photocatalytic activity. In order to control the concentration of oxygen vacancies, Wang et al. used an appropriate amount of Nb^5+^ (r = 0.064 nm) to replace Ti^4+^ (r = 0.061 nm) ions to balance the negative charge brought by N-doped Sr_2_TiO_4_. The results show that Nb/N co-doped layered perovskite Sr_2_TiO_4_ exhibits optimal photocatalytic degradation performance for tetracycline when the doping amount of Nb^5+^ is 2% [32]. In the study of Sun et al., the DOS calculation results show that the conduction band minimum (CBM) is dominated by Ti 3d orbitals, while the valence band maximum (VBM) mainly includes O 2p and N 2p hybrid orbitals [26]. The hybridization of N 2p and O 2p orbitals will increase the VBM, thus reducing the band gap of La/N co-doped Sr_2_TiO_4_. However, La has no contribution to CBM and VBM near the Fermi level and can be used as a balancer to balance the charge difference caused by O/N substitution. Based on the fact that the hybridization of N 2p and O 2p orbitals can increase the maximum valence band of Sr_2_TiO_4_ and La can balance the charge difference caused by N/O substitution, Sun et al. achieved strong visible light absorption up to 650 nm by changing the La/N content in Sr_2−x_La_x_TiO_4-y_N_y_ (0 ≤ x ≤ 0.5).

**Figure 10:**
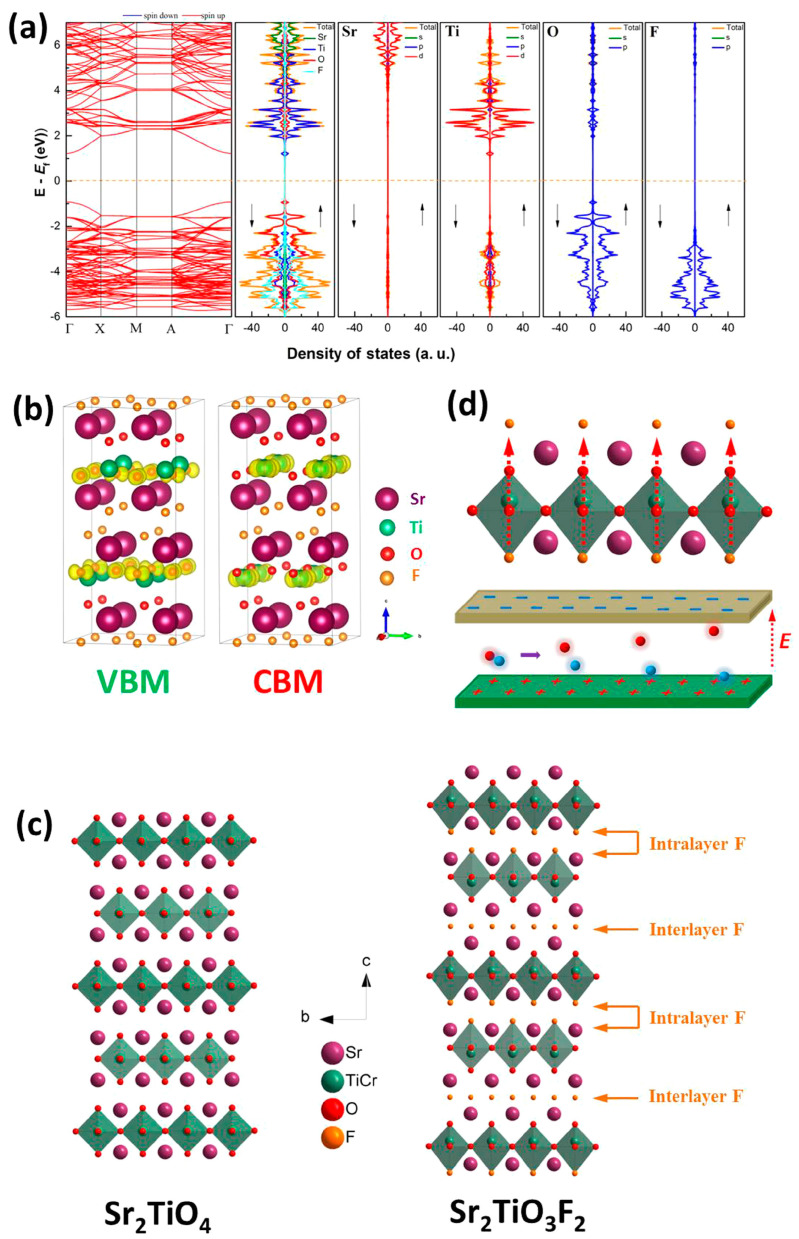
(**a**) Calculated band structures, the total density of states, and partial density of states of constituent elements of Sr_2_TiO_3_F_2_, and Fermi level is marked by dotted orange line; (**b**) density contour maps of Sr_2_TiO_3_F_2_ at VBM and CBM; (**c**) crystal structure of Sr_2_TiO_4_ and Sr_2_TiO_3_F_2_ projected along (100) direction; (**d**) schematic illustration of a built-in electrical field across the single TiO_6_ octahedron layer (upper image) and its impact toward the separation of photogenerated electron–hole pairs (lower image), direction of electric field is indicated by red dotted arrow, red and blue balls refer to photogenerated electrons and holes, respectively (for interpretation of the references to color in this figure legend, the reader is referred to the web version of this article) [62].

In addition, the ionic radius of F^−^ is similar to that of O^2−^; thus, F^−^ replacing O^2−^ can better adapt to the original crystal structure in the lattice to reduce the lattice distortion. Moreover, F has the highest electronegativity and can introduce different electronic properties during the doping process. Yu et al. modified Cr-doped Sr_2_TiO_4_ with fluorine to obtain a new compound Sr_2_Ti_0.95_Cr_0.05_O_3_F_2_, which has two types of fluorine in the structure of the layer and the interlayer (Figure 10) [62]. Due to the non-uniform distribution of F atoms on both sides of the perovskite layer, the charge disproportionation leads to a strong built-in electric field. This unique structural feature is very beneficial to charge dissociation, because it breaks the coplanar settlement of the minimum conduction band and the maximum valence band while maintaining the 2D charge transport characteristics. Sr_2_Ti_0.95_Cr_0.05_O_3_F_2_ exhibits extremely high photocatalytic hydrogen production activity, achieving an apparent quantum efficiency (AQE) of up to 1.16% at 420 ± 20 nm.

#### 2.2.4. Composites Form Heterostructures

**Figure 11:**
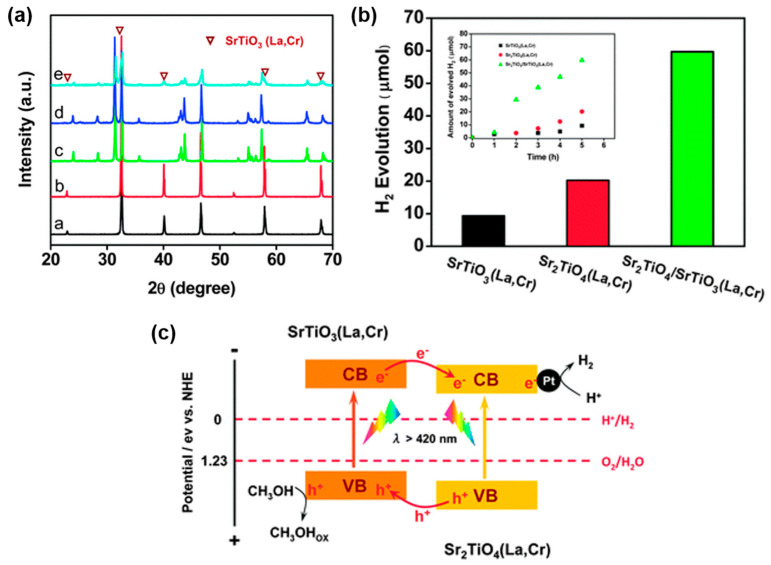
(**a**) X-ray diffraction patterns of the as-prepared samples: a SrTiO_3_, b SrTiO_3_ (La,Cr), c Sr_2_TiO_4_, d Sr_2_TiO_4_ (La,Cr), and e Sr_2_TiO_4_/SrTiO_3_ (La,Cr), (**b**) photocatalytic H_2_ production activities of the initially synthesized SrTiO_3_ (La,Cr), Sr_2_TiO_4_ (La,Cr) and Sr_2_TiO_4_/SrTiO_3_ (La,Cr) composite samples ([Cr]/([Ti] + [Cr]) = 0.05, [La]/[Cr] = 1.00, molar ratio). Inset: the time course of H_2_ evolution, (**c**) schematic band structure of La and Cr co-doped Sr_2_TiO_4_/SrTiO_3_ and its mechanism for H_2_ production under visible light irradiation [64].

Compared with single-component photocatalysts, designing material recombination to form heterojunctions may be an ideal method to achieve effective charge separation to enhance the photocatalytic activity of water splitting (Table 3) [63]. Jia et al. prepared a Sr_2_TiO_4_/SrTiO_3_ (La, Cr) heterojunction composite photocatalyst by a facial in situ polymerization approach (Figure 11a). In the composite photocatalyst, photogenerated electrons and holes tend to migrate from SrTiO_3_ (La, Cr) to Sr_2_TiO_4_ (La, Cr) and from Sr_2_TiO_4_ (La, Cr) to SrTiO_3_ (La, Cr), respectively (Figure 11b). This band structure can promote the charge transfer and separation driven by the small potential difference between the two components, making the composite heterojunction photocatalyst exhibit excellent photocatalytic activity (Figure 11c) [64].

Based on previous studies, developing composite photocatalysts by coupling n-type and p-type semiconductors is a promising strategy. It can improve its photocatalytic performance through appropriate band bending and high charge mobility and light absorption efficiency [65,66,67,68]. As a typical p-type semiconductor, Bi_5_O_7_I can provide more photo-excited holes for oxidation, showing a relatively more positive valence band level than other bismuth oxyiodides [69,70]. Moreover, Bi_5_O_7_I is obtained from the competition between I− and OH^−^ ions under alkaline conditions. This leads to a high Bi/I ratio and drives Bi ions to diffuse into the lattice of Sr_2_TiO_4_. In addition, Bi_5_O_7_I has the same layered structure as Sr_2_TiO_4_, which is conducive to the infiltration and substitution of Bi ions into the lattice of Sr_2_TiO_4_ [27,71]. In view of the above, Hu et al. synthesized a Bi_5_O_7_I/Sr_2_TiO_4_ composite via an in situ wet-chemical method [27]. Due to the p-n junction in the Bi_5_O_7_I/Sr_2_TiO_4_ heterostructure, a space charge region is formed at the interface, resulting in effective charge separation. In addition, Ag loading on the surface of the Bi_5_O_7_I/Sr_2_TiO_4_ heterostructure induces localized surface plasmon resonance, which increases the absorption of visible light and promotes the photocatalytic degradation of the MO solution [71].

**Table 3:** Properties of Sr_2_TiO_4_ compositing with differentiation.

Composites	Reactions	E_g_ (eV)	Ref.
Bi_5_O_7_I/Sr_2_TiO_4_	degradation of MO	3.15	[27]
Sr_2_TiO_4_/SrTiO_3_(La,Cr)	hydrogen evolution	2.52	[64]
Ag@Sr_2_TiO_4_/Bi_5_O_7_I	Photoelectrochemical	3.10	[71]

## 3. Conclusions

In this concise minireview, we have encapsulated the intrinsic characteristics and optimization strategies including surface modification and interface engineering for Sr_2_TiO_4_ layered perovskite. By producing element defects on the surface of Sr_2_TiO_4_ through different approaches, the light utilization ability and charge separation efficiency can be effectively improved, thus promoting the photocatalytic activity. The surface deposition of metal cocatalysts could generate an LSPR effect, thereby promoting photocatalytic performance. Morphology control over Sr_2_TiO_4_ for photocatalysis has also been concluded with detailed synthetic strategies. Moreover, Sr_2_TiO_4_-based composites such as the p-n junction have been discussed. Useful strategies for designing RP perovskites Sr_2_TiO_4_ in photocatalysis have been provided to provide helpful guidance for future investigations.

In the future, it is necessary to explore Sr_2_TiO_4_ in several directions to develop its greater value and application potential. First, the mechanistic understanding of photocatalytic processes involving Sr_2_TiO_4_ needs to be strengthened in order to further improve its photocatalytic performance. For example, some in situ characterization techniques can also be used to monitor its structure change during photocatalytic reactions to gain a deeper understanding of its photocatalytic process, providing practical guidance for optimizing its structure. Secondly, the investigation of Sr_2_TiO_4_’s exfoliation technology is still in its infancy; breakthroughs in this area could unlock new avenues for the material’s application in thin films or as a support for other catalytic materials. Furthermore, it can also be intercalated to make the interlayer material type adjustable after stripping, which will profoundly impact the photocatalytic field. Lastly, the scalability and economic viability of Sr_2_TiO_4_ production methods will be crucial for its widespread adoption in industrial applications. Research into more sustainable and cost-effective synthesis routes is, therefore, of paramount importance. In conclusion, this minireview has provided a solid foundation for future research endeavors. By building upon the current body of knowledge and exploring new avenues of investigation, the Sr_2_TiO_4_ has the potential to become a leading semiconductor in the field of photocatalysis, particularly for environmental remediation applications and energy utilization.

## Figures and Tables

**Table 1:** Different morphologies of Sr_2_TiO_4_.

Synthesis Methods	Calcination Temperature	Morphology	Size	Ref.
Polymerized complex	900 °C	nanoparticles	200 nm	[21]
sol–gel	700 °C	cubic rod-like particles	3–6 μm	[27]
sol–gel	1000 °C	nanoparticles	43 nm	[32]
sol–gel	900 °C	nanoparticles	185 nm	[44]
polymeric and hydrothermal	1000 °C	nanosheets	350 nm	[49]
sol-precipitation	1100 °C	spheroidal nanoparticles	5 μm	[50]
sol-precipitation	1100 °C	flat particles	1.9–5.7 μm	[50]
co-precipitation	1100 °C	circular particles	4.7 μm	[50]
mechanochemical	1100 °C	rough particles	2–10 μm	[50]
sol–gel	1000 °C	nanoparticles	248 nm	[51]

**Table 2:** Properties of Sr_2_TiO_4_ doping with differentiation.

Doping Elements	Doping Site	Doping Content	Reactions	E_g_ (eV)	Ref.
Ag	A	2.5 at%	hydrogen evolution	3.05	[44]
F	O	3 at%	hydrogen evolution	3.20	[51]
La/Rh	A/B	1.5 at%/3 at%	hydrogen evolution	2.40	[21]
La/N	A/O	10 at%/-	water splitting	2.20	[26]
La/Fe	A/B	1.5 at%/3 at%	hydrogen evolution	2.80	[31]
Nb/N	B/O	-	degradation of TC	2.95	[32]
Cr/F	B/O	5 at%/40 at%	hydrogen evolution	2.48	[62]
